# Exposures Resulting in Safety and Health Concerns for Child Laborers in Less Developed Countries

**DOI:** 10.1155/2016/3985498

**Published:** 2016-06-12

**Authors:** Derek G. Shendell, Saisattha Noomnual, Shumaila Chishti, MaryAnn Sorensen Allacci, Jaime Madrigano

**Affiliations:** ^1^Rutgers School of Public Health (SPH), Center for School and Community-Based Research and Education (NJ Safe Schools Program), New Brunswick, NJ 08854, USA; ^2^Department of Environmental and Occupational Health, Rutgers SPH, Piscataway, NJ, USA; ^3^Exposure Measurement and Assessment Division, Environmental and Occupational Health Sciences Institute, Rutgers Robert Wood Johnson Medical School, Piscataway, NJ 08854, USA; ^4^Rutgers Center for Green Building, Edward J. Bloustein School for Planning and Public Policy, Rutgers, The State University of NJ, New Brunswick, NJ 08901, USA; ^5^School of Arts and Sciences, Rutgers, The State University of NJ, New Brunswick, NJ 08901, USA

## Abstract

*Objectives*. Worldwide, over 200 million children are involved in child labor, with another 20 million children subjected to forced labor, leading to acute and chronic exposures resulting in safety and health (S&H) risks, plus removal from formal education and play. This review summarized S&H issues in child labor, including forced or indentured domestic labor as other sectors of child labor. Specifically, we focused on exposures leading to S&H risks.* Methods*. We used PubMed, Scopus, Science Direct, and Google Scholar. References were in English, published in 1990–2015, and included data focused on exposures and S&H concerns of child labor.* Results*. Seventy-six journal articles were identified, 67 met criteria, 57 focused on individual countries, and 10 focused on data from multiple countries (comparing 3–83 countries). Major themes of concern were physical exposures including ergonomic hazards, chemical exposure hazards, and missed education. Childhood labor, especially forced, exploitative labor, created a significant burden on child development, welfare, and S&H.* Conclusions*. More field researche data emphasizing longitudinal quantitative effects of exposures and S&H risks are needed. Findings warranted developing policies and educational interventions with proper monitoring and evaluation data collection, plus multiple governmental, international organization and global economic reform efforts, particularly in lower-income, less developed countries.

## 1. Introduction

Worldwide, over 200 million children are involved in child labor, and over 20 million children are subjected to forced labor [1, 2]. These children are thus removed from formal education, play, and other opportunities for healthy social and personal development. Globally, children can be found working in various industries like agriculture, construction, fishing, mining, small-scale businesses, the informal sector, and manufacturing for export and domestic sales as well as in homes for child care of family members, assisting in cleaning and cooking, and so forth [3]. These children are often involved in producing consumer products and food used by citizens in their communities as local subsistence or services but also to supply global markets. Therefore, in their daily work, children may be exposed to acute and chronic safety and health (S&H) risks due to multiple exposure agents indoors and outdoors (and in semienclosed areas).

Internationally, the fundamental conventions of the International Labor Organization (ILO)—to which ratifying member countries are then automatically bound to monitoring and enforcing independently—include Convention 138 of 1973 (especially Articles 1–3) and Convention 182 of 1999 (especially Articles 2, 3, and 7) [4, 5]. The relevant articles of Convention 138, entered into force June 19, 1976, are “Minimum Age for Admission to Employment,” and relevant articles of Convention 182, entered into force November 19, 2000, are “Prohibition and Immediate Action for the Elimination of the Worst Forms of Child Labour” [4, 5]. These two ILO Conventions were founded on worker and child (under age 18) human rights plus moral, social, and S&H related concerns [4, 5]. Both ILO Conventions also relate to the United Nations Convention on the Rights of the Child, specifically its Article 32 [6]. Unfortunately, evidence has suggested problems persist worldwide, especially in lower-income, less developed countries (LDCs).

In the United States (US), resulting from the documented child labor reform movement of the late 19th century, US policy change was the result not only of moral and economic forces, but also from a growing body of scientific evidence on the general susceptibility and vulnerability of children to S&H hazards/exposures and adverse health outcomes, respectively, and the scale of the problems posed by child labor [7].

Therefore, the main objective of this literature review was to summarize exposure and S&H risk-related issues in child labor in LDCs. The concurrent goal was to increase working knowledge and heightening awareness of child labor S&H issues among readers, particularly younger and mid-career professionals entering public health directly from university and/or healthcare and policy-related fields. It is essential for public health researchers and practitioners to obtain a basic understanding of the global magnitude of child labor.

## 2. Materials and Methods

We conducted a review of peer-reviewed journals via the US National Library of Medicine's PubMed, Scopus, ScienceDirect, and Google Scholar as well as weekly updates from SafetyLit (specifically the “adolescent health” and “occupational health issues” subcategories), a citation/abstract indexing service of the World Health Organization (WHO) via San Diego State University. The aforementioned subcategories also covered child street labors and child labor-related street-based activities. Keywords used included child labor, young workers, youth workers, young adult workers, and street vendors. Additional references were reviewed for their potential theoretical, economic, and structural explanations for persistence and locational patterns of child labor-focused industries. Sources included indices in the humanities and social sciences (e.g., Project MUSE, Historical Abstracts, and Alternative Press Index) economics and business (e.g., EconLit, Business Source Premier), and education (ERIC). To be included, references had to be available in the English language; be published in the final decade of the 20th century or afterwards (1990–2015); be focused primarily on child labor—among school-aged children ages 5–18—and its S&H risks primarily due to physical, chemical, radiological, and/or biological exposures; and include data on exposures leading to S&H risks and outcomes. It must be noted how articles addressing adolescents and young adults up to age 21, where identified, remained directly relevant and thus included in the search results. Because of a focused interest in peer-reviewed publications, we immediately excluded nonprofit/nongovernmental organization advocacy articles published only online—not a journal paper and not a final report/formal white paper—and articles which covered multiple emerging issues in specific sectors, for example, electronic waste or agriculture, where children may work in small-scale, family-oriented community-based settings. We also excluded website-based descriptions of—and available final reports on—grants and cooperative agreements of the US Department of Labor-International Labor Bureau [1, 2].

In summary, 76 articles were identified. Bibliographic information was organized into EndNote then Excel. It must be noted how even if we searched 1990–2015, most identified papers (Tables 1 and 2) were published 2001-present; only three were published before, 1992–1997. We then excluded eight of the 76 as not being peer-reviewed journal articles: two Human Rights Watch reports from 2002 and 2004; a 2002 World Bank Policy Research Working Paper 2897 by Gustafsson-Wright and Pyne; an ILO report from 2013; a 2005 report by DeBoer via Terre des Hommes, The Netherlands; a report by Ersado in 2005, which was a precursor to a peer-reviewed journal article included in the final citation list; a 2005 working paper by Rogers and Swinnerton; and a 2005 CUDARE working paper by Anokhi and Elisabeth. One peer-reviewed paper published in 2006 by Pinzón-Rondóna et al. in Latin America was also excluded because the full article was not in English. The remaining 67 articles were reviewed and summarized, with a specific focus on industry and exposure agents (S&H hazards/risks) of concern.

## 3. Results

Of the 67 journal articles included in this focused literature review (Tables 1 [Table tab2]–3), 57 pertained to individual countries published between 1992 and 2014 (Tables 1 and 3) across the six WHO administrative regions (Table 1). They included 47 LDCs, four central/eastern European countries (Albania, Kazakhstan, and Turkey (three papers)) and one African country (South Africa) in economic transition; three on industrialized nations with substantial indigenous populations (Australia, New Zealand, and Finland); and two on US low-income, minority agricultural populations related to immigration and other reemerging “cross-border” topics with Mexico (and potentially other Central American nations) presenting substantial political and S&H issues. These 57 papers concerning individual countries can first be stratified by industry and then exposure agents of concern. It should be noted how one article incorporated two case studies about Kazakhstan and Mali although the pertinent Mali data were from surveys completed by child worker parents/caregivers [3]. The other 10 papers, published between 2005 and 2011, focused on multiple countries with comparisons covering 3–83 countries located in one, two, three, or five of six WHO administrative regions (Tables 2 and 3) [8].  Table 3 summarized those studies on street-based activities/industries which also reported data on exposures (specific hazards/risks) and health effects/outcomes.

The main results of these studies were summarized below.

Twenty-five of 57 papers focused on specific countries pertained to agriculture or selling and vending agricultural products like food on streets (also see Table 3). Specifically, the articles covered agriculture in general or specific crop-based industries, for example, cocoa in Ghana and tobacco in Kazakhstan or Malawi [3, 9, 10]. One paper from Finland discussed the impact of agricultural and nonagricultural labor on adolescents as young workers with income with respect to excessive alcohol consumption [11]. Of the 16 other papers related to agricultural work in general, six papers also related to nonagricultural, nonmining work in various manufacturing and export sales industries like carpet weaving and shoes [12–17]; four papers also related to nonagricultural, nonmining work, and domestic work like child care, cooking, and cleaning [18–21]; and one paper also related to both nonagricultural work and mining activities [22]. As previously noted, Amon et al. also cited data from a case study in Mali on artisanal small-scale gold mining [3]. Another paper by Banerjee discussed both agricultural and domestic work in West Bengal, India [23]. The other four papers only pertained to agricultural work [24–27]. It should be noted how Hendricks placed emphasis on racial and ethnic minority subpopulations working on minority subpopulation owned farms [24], and Corriols and Aragón focused on acute poisonings due to the use of various chemical pesticides [26]. The majority of adverse impacts reported in these 25 papers covering agriculture or agriculture and another sector were physical injuries and absence from school. It must be noted how these children may not just be absent from school from time-to-time; that is, they may miss school altogether, for reasons ranging from child labor, family circumstances including poverty and caretaker responsibilities of younger siblings or sick relatives, lack of access to school supplies, and so forth. Education is a component of socioeconomic status (SES), and lower SES is well known to be generally associated with adverse health outcomes. Other adverse health outcomes less frequently reported included skin irritation and neurotoxicity due to the use of chemical pesticides, physical and verbal abuse, malnutrition and/or undernutrition, and, in some cases, mortality.

The other papers—32 of 57—focused on child labor in nonagricultural industries.

Five of the papers explored mining, specifically mining involving stone polishing and silicosis in Brazil [28]; small-scale or artisanal mining, whether for various minerals or gold [29–31]; or stone quarries [32]. Similar adverse health outcome categories as in the agricultural sector were reported in the mining sector, including injury, abuse, mortality, and loss of education.

Eight of the papers explored physical/ergonomic and/or chemical hazards (exposures) during nonagricultural, nonmining work in various manufacturing and export sales industries [33–42], and another eight of the papers were on specific manufacturing and/or export sales industries [43–50]. The carpet weaving industry was highlighted in India by Das et al. [43] and Joshi et al. [44], in Iran by Choobineh et al. [45], and in Pakistan by Awan et al. [46]. Gurcanli focused on the construction industry in Turkey [47]. Tiwari wrote about the shoe manufacturing industry, including rubber and leather soles, in India [48]. Mitra examined the small-scale leather industry in Calcutta, India [49]. Harari and Cullen reported on a small, cross-sectional study of children working in the home-based or “cottage” industry of ceramic roof tile production in Ecuador [50]. In summary, five studies reported on respiratory symptoms, including coughing from illnesses like respiratory infections [35, 43–46], and three studies reported on ocular health (eyestrain) due to specific tasks characteristic of these manufacturing industries [38, 45, 47]. Specific manufacturing tasks of concern highlighted were the production of hand-woven carpets in Iran [43] and of shoes in India [46].

Six other papers focused on the potential impact of nonagricultural, nonmining work and domestic work. Contributing factors and outcomes of concern were a child's educational attainment and school attendance in rural Cambodia or in rural Bangladesh [65, 66]; or the explicit role of household/family poverty [67]; or the recruitment of children into work and their later reintegration into society in Albania [68]; or the perceptions of work and social aspirations of children in Ibadan, Nigeria [69]. In these five studies, in summary, the major exposure of concern was social stress, typically as related to lower family SES, and the major outcome of concern was loss of education due to absence from school and/or some form of abuse [65–69]. In addition, Schlick et al. covered nonagricultural, nonmining work and domestic work in Cusco Province of Peru [70]. In this study, physical hazards resulting in falls were the greatest reported concern [70]. It should also be noted how in two of these five studies gender showed varying results—there were differences reported by Naeem et al. in Pakistan [67], where most girls—outnumbered compared to the boys 1 : 5 in the study sample—were reported to work as domestic servants or as pickers through garbage at dumpsites, but no statistically significant differences were reported by Shafiq in Bangladesh [66].

In addition, seven of the 52 papers focused on nonagricultural fields and nonmining industry work, in terms of street-based activities related to children's quality of life as well as exposures (hazards/risks) and adverse health effects/outcomes (Table 3). Furman and Laleli reported that lead hair concentrations among child street vendors in Istanbul, Turkey, were five times greater than a control group, especially among those who were in high density traffic areas [62]. Work by children conflicting with schooling was reported in three studies: Baron in the informal sector in Mexico [60]; Bromley and Mackie among traders in Cusco, Peru [61]; and Gharaibeh and Hoeman in garages in Jordan [63]. Some children dropped out of school because they needed to work for money to support their families. In addition, Bromley and Mackie noted how the children chose to work because work can lead to empowerment and increased self-esteem [61]. Nevertheless, these children experienced chemical exposures as well as psychological and physical assaults, that is, verbal and sexual abuses, especially the girls and younger children. Working on streets was related to injuries (i.e., scratches, cuts/lacerations, burns, car accidents, sprains, amputations, and paralysis) as reported by Baron [60] and Pinzón-Rondóna et al. [53]. Baron also reported how work-related injuries among children were more severe than non-work-related injuries, and commuting to work likely caused many of those injuries [60]. Furthermore, results suggested gender influenced work-related injuries among children; males had more injuries resulting from construction-related jobs while female had more injuries from work in stores, markets, and restaurants [60]. Pinzón-Rondóna et al. later reported how for each additional ten hours per week of children's work on streets, there was an increased prevalence of abuse in working areas as well as an increased prevalence of occupational injuries [54]. Mondal et al. studied the sociomedical profiles among child workers along railways in India; about 1-in-4 children had some forms of handicap and/or illness, that is, respiratory tract infections or RTI, eczema, diarrhea, and abdominal pain [64].

Three other nonagricultural papers were conceptual in nature, without data on exposures and adverse outcomes. Invernizzi covered the analysis of children's work related to the socialization process, which was complementary to exploitation [71]. Estrada and Hondagneu-Sotelo studied perspectives among Latino immigrant children engaged in street vending [72]. Rother explained the contextual factors and externalities (i.e., unintended consequences) of work involving street pesticide sales and use among children [73].

Finally, two other papers examined male working adolescents in Jordan. Authors reported associations (correlations) between maternal attributes, smoking status, monthly income from work (child and family), age at start of work, and length of time at work with weight and height (and resulting body mass index calculations). Results suggested growth-related anthropometric measures of working males were negatively impacted by work [74, 75].

Ten papers with an international (multicountry or global) scope were published 2005–2015. Eight papers compared some LDCs to each other or to an industrialized nation; one paper was an economic modeling analysis pertinent to LDCs with rapidly growing economies; and one paper capitalized on larger cross-sectional multicountry population-level data sets. Ersado covered three LDCs from three different WHO administrative regions—Nepal, Peru, and Zimbabwe. They conducted comparative analyses of decisions made about going to work versus attending school [52]. Chaudhuri focused on potential advantages and disadvantages of labor market reforms affecting the reported incidence of child labor within developing/emerging economies [76]. Levison and Langer focused on children working as domestic servants a common occupation for girls in some countries, in six countries in the WHO region of the Americas—Argentina, Brazil, Chile, and Colombia in South America and Costa Rica and Mexico in Central America—and suggested they were sometimes better off than nondomestic servant child laborers with respect to increased school enrollment [56]. Gamlin et al. surveyed over 3000 children, nearly half involved in domestic work, to examine physical exposures and psychological effects of such work in six countries spanning four WHO regions—Peru, Costa Rica, Tanzania, Togo, India, and the Philippines—with the most associated S&H risks reported in India and Togo [57].

Webbink et al. investigated social determinants of children performing housework and family-based small business work in 16 different countries across two of the six WHO administrative regions, Africa and Southeast Asia (see Table 2). These “hidden” forms of child labor were reported as common—about 30% of African children and about 10% of Asian children were engaged in them ≥15 hours per week. Socioeconomic factors, such as household wealth and maternal education, were associated with the decreased likelihood of a child engaging in these forms of labor. Improved household infrastructure, such as electricity and water, were also associated with a significant reduction in hours spent on housework by children [58].

Whetten et al. covered five countries, two in Southeast Asia (Cambodia, India) and three in Africa (Ethiopia, Kenya, and Tanzania). The focus of this analysis was child labor and work characteristics among orphaned children in these selected lower-income (Cambodia, Ethiopia, and Tanzania) and middle-income (India, Kenya) LDCs. Female orphans and those from poorer households had increased odds of being engaged in child labor. This study also suggested working ≥28 hours per week was associated with decreased school attendance [55].

Rohlman et al. compared two LDCs, Egypt and Lebanon, to the US. Specifically, this paper summarized the main findings on chemical exposures and risks to young worker health reported and discussed at “Using Epidemiology and Neurotoxicology to Reduce Risks to Young Workers,” a session within the concurrent June, 2011, 13th International Neurotoxicology Association Meeting and 11th International Symposium on Neurobehavioral Methods and Effects in Occupational and Environmental Health, Xi'an, China [51].

Roggero et al. obtained prevalence year 2000 data from 83 countries across five of the six WHO administrative regions (see Table 2) for comparative cross-sectional statistical analyses of existing health-related outcome indicators due to child labor, that is, children aged 10–14 who worked according to the World Bank. Outcomes of interest among children aged 5–14 and adults, by gender, included variables related to child and adult morbidity and mortality data; population-level nutritional status as undernourishment (i.e., insufficient total calorie intake); and the prevalence of infectious diseases like HIV/AIDS, malaria, and others related to poor sanitation, dangerous work, and/or nutritional status. Child labor was associated with adolescent mortality [59].

Several papers were also reviewed for their contribution to explaining the persistence and resilience of child labor practices. Overall, distinction may be made between exploitive and beneficial child labor practices. There were differences in the effects of global economic and cultural participation on child labor practices, and they varied by region and industrial sectors.

For example, Clark noted how children in rural areas were most likely to be active in labor, in part because of the poor proximity of quality schooling and lack of enforcement, ultimately leading to a reduction in skill preparation for future success. He also referenced other works documenting persistent poverty, lax enforcement, and isolation of families from global economic and cultural trends contributing to the persistence of child labor activities. Furthermore, he noted how trade has had a greater effect on reductions in child labor than foreign investment [77].

The family or household status also influenced labor-related effects. Pinzón-Rondóna et al. discussed how the economic exploitation of children as beggars by parents for family basic needs was more likely to occur among children living with their mothers in four Latin American cities: Bogotá, Colombia; Lima, Peru; Quito, Ecuador; and São Paulo, Brazil [54]. Parker and Overby suggested child labor and health might be explained by a model integrating economic development, education, and labor regulation; the children lacking financial resources and education, and suffering from impaired growth or development, tended to engage in work [78].

Phillips, Bhaskaran, Nathan, and Upendranadh also noted child labor practices could not be explained entirely by household or individual level factors, but rather they involved systems analysis including how socially embedded commercial processes are associated with modern global production processes [79]. Thus, while global product networks can lead to economic improvements and labor protections in some regions, in other areas they have the opposite effect: that is, government structures have supported the deregulation of the private industry and constraints on labor rights including those of children.

Doytch et al. suggested how despite the overall reduction in child labor rates since the 1960s, analyses of child labor trends and conditions have suggested important differences by sector and region have persisted, with both positive (e.g., agricultural) and negative (i.e., manufacturing) associations with foreign direct investment (FDI). For example, positive links have been found between FDI and West Africa's copper, gold, and cocoa belts as well as between FDI and mining sector child labor in Mali, an especially dangerous industry for children and where the closer the schools located to the mines, the higher the number of dropouts from school. These authors pointed to the associations of extensive and complex supply chains of large, multinational corporations with child labor abuses and thus suggested improved monitoring and information disclosure, the reform of which can add to costs. Government complicity with corporate labor exploitation, for example, Kazakhstan, where migrant children were hindered from registering for school, was also seen an important contributor to labor abuses [80].

## 4. Discussion

Overall, this focused literature review suggested how child labor remains a significant public health issue and welfare burden worldwide, even with multiple international conventions [4–6] and thus suggests these conventions, particularly ILO Convention 182, could be revised [81].

Globally, children are exposed to numerous physical agents, including extreme heat or cold and poor ergonomics, as well as psychological stressors in a wide range of industrial sectors. The main sectors included agricultural work pre- and postharvest, mining, domestic work, and trades like carpet weaving, shoe making, and construction. Chemical and biological exposure agents (hazards) reported with published data within this review were summarized in categories (Table 4).

As a consequence of these working environments, children experience various physical injuries and illnesses, ranging from mild symptoms to severe disability, sometimes even death. Child labor, and forced labor in particular, can also manifest in psychological trauma due to psychosocial stressors—an emerging category of coexposure agents—like verbal and physical abuses, and prolonged absence from school, which is especially detrimental if prior to finishing primary school. Adverse outcomes were compounded, across gender, by family poverty.

Combating exploitative child labor more effectively would seem to require an improved understanding of the magnitude and nature of the problems posed to S&H and the patterns under which they exist and are likely to continue to negatively affect the child's physical, mental, social, and spiritual health [6]. A complete characterization of the types of hazards children are exposed to and resulting morbidity, disability, and mortality has proved difficult because comprehensive data sources have not been available, particularly in LDCs. Recently, however, the ILO Statistical Information and Monitoring Program on Child Labor (SIMPOC) has partnered with over 40 countries to study occupational injury and illness in children; one of those surveys included, and reviewed in the present paper, was the Philippine Survey of Children [15]. Although there were some limitations, including its inclusion of only nonfatal injuries, potential for inaccuracies resulting from child respondents, and its cross-sectional nature, it remains one of the few surveys to provide national estimates of occupational illness and injury among children in a larger LDC.

It should be noted how most of the studies cited in the present review were based on cross-sectional surveys with limited sample sizes, including interview questionnaires with workers or parents/guardians/caregivers of workers (especially if children), and population-level estimates for a given year. These are not prospective cohort studies with repeated measures. Without the availability of larger, longitudinal follow-up studies, the long-term health implications and potential gender disparities of childhood labor—except for missed school days—cannot yet be accurately characterized. Therefore, the overall global health burden of disease due to child labor likely remains underestimated. More research is needed to better understand the extent to which child labor practices are associated with poor quality of life indicators and the roles complex global economic, government, and other relationships play in continuing exploitation of children. Furthermore, research to compare similar industries across different geographic and economic contexts would help better define the problem as industry-wide or as a function of industry context and specific attributes supporting the problem.

Studies cited in this review consistently documented how children of poor families/communities, that is, of lower SES, are at increased risk of being involved in child labor [14, 20, 23, 67, 70, 76] and therefore potentially missing out on education at school or at home, but not always [12, 17, 18, 21, 50, 65, 66]. Consequently, while labor policy and enforcement are critical to addressing this global problem, they cannot eradicate it in isolation—the broader problem of poverty must also be addressed, and the capacity to deliver compulsory primary education through age 15 [4, 5] must be ensured. Family size and number of children in a family have also been shown to increase the likelihood of a child being engaged in labor [14, 20]. For example, Srivastava described how the Indian government is taking a multidisciplinary approach to childhood labor, including working with nonprofits and through legislative action to increase education, improve the economic condition of the families, and offer primary healthcare checkups for children [19].

## 5. Conclusion

This review can serve readers—especially younger and mid-career professionals entering public health directly from university and/or healthcare and policy-related fields—to increase their working knowledge and awareness of, and potentially change attitudes about, exposures to chemical, physical, biological, and psychosocial (social and psychological) agents or factors resulting in safety and health (S&H) risks in child labor, particularly with respect to lower-income, less developed countries (LDCs). With over 200 million children involved in child labor and another 20 million or more children subjected to forced labor worldwide, child labor creates a significant burden on the development, welfare, and overall health of children. Given the limited peer-reviewed work identified, more field research on child labor, with longitudinal quantitative measures on exposures and S&H risks, are needed in lower-income LDCs. These projects would serve as improvements to past and ongoing cross-sectional annual population-based national surveys and specific subpopulation assessments. In addition, results of research to date as summarized in this review warrant more policy and educational interventions with proper monitoring, enforcement, and evaluation, including multiple governmental and international organization efforts, in low-income LDCs.

## Figures and Tables

**Table 1:** Number of studies published about child labor safety and health risks, by country and by WHO region.

Country of specific focus^a^	Number of articles
Albania	1
Australia	1
Bangladesh	1
Brazil	3
Cambodia	1
Colombia	1
Ecuador	1
Egypt	1
Ethiopia	1
Finland	1
Ghana	2
India	10
Indonesia	1
Iran	2
Jordan	3
Kazakhstan	1
Lebanon	3
Malawi	1
Mali	1
Mexico	1
Nepal	2
New Zealand	1
Nicaragua	1
Nigeria	2
Pakistan	2
Peru	2
Philippines	2
Sierra Leone	1
South Africa	1
Thailand	1
Turkey	3
United States (immigrants)	2

WHO region: Africa	9
WHO region: The Americas	11
WHO region: Eastern Mediterranean	13
WHO region: Europe	5
WHO region: Southeast Asia	13
WHO region: Western Pacific	6

^a^Multicountry studies identified in journals (*n* = 10) excluded here but in references list and discussed in text and/or Tables 2 and 3.

**Table 2 tab2:** Studies published on child labor safety and health risks which included data on multiple countries and World Health Organization (WHO) organizational regions (*n* = 9 of 10; Chaudhuri (2009) not listed, was economic model).

Reference	WHO regions	Number of countries	Countries included
Rohlman et al. (2012) [51]	The Americas, Eastern Mediterranean	3	Egypt, Lebanon, United States

Ersado (2005) [52]	Africa, The Americas, Eastern Mediterranean	3	Nepal, Peru, Zimbabwe

Pinzón-Rondóna et al. (2009, 2010) [53, 54]	The Americas	4	Brazil, Colombia, Ecuador, Peru

Whetten et al. (2011) [55]	Africa, Southeast Asia	5	Cambodia, Ethiopia, India, Kenya, Tanzania

Levison and Langer (2010) [56]	The Americas	6	Chile, Argentina, Mexico, Costa Rica, Brazil, Colombia

Gamlin et al. (2015) [57]	Africa, The Americas, Southeast Asia, Western Pacific	6	Costa Rica, India, Peru, Philippines, Tanzania, Togo

Webbink et al. (2012) [58]	Africa, Southeast Asia	16	Bangladesh, Burundi, Central African Republic, Cote d'Ivoire, Gambia, Ghana, Guinea Bissau, Sierra Leone, Togo, Malawi, Mauritania, Somalia, Syria, Thailand, Vietnam, Yemen

Roggero et al. (2007) [59]	Africa, The Americas, Eastern Mediterranean, Southeast Asia, Western Pacific	83	Algeria, Angola, Bangladesh, Belize, Benin, Bolivia, Botswana, Brazil, Burkina Faso, Burundi, Cambodia, Cameroon, Chad, Chile, China, Congo, Colombia, Cote d'Ivoire, Costa Rica, Democratic Republic of Congo, Dominican Republic, Egypt, Ecuador, El Salvador, Eritrea, Gabon, Gambia, Ghana, Guatemala, Guinea, Guinea Bissau, Haiti, Honduras, India, Indonesia, Iran, Iraq, Jamaica, Jordan, Kenya, Laos, Liberia, Libya, Lesotho, Madagascar, Malaysia, Malawi, Mali, Mexico, Mongolia, Mozambique, Morocco, Myanmar, Namibia, Nepal, Nicaragua, Niger, Nigeria, Oman, Pakistan, Panama, Papua New Guinea, Paraguay, Peru, Philippines, Rwanda, Senegal, Sierra Leone, Solomon's Islands, Sri Lanka, Sudan, Swaziland, Syrian Arab Republic, Uganda, Uruguay, Tanzania, Venezuela, Vietnam, Thailand, Togo, Yemen, Zambia, Zimbabwe

**Table 3:** Studies focused on street-based activities and industries involving children and young adults, which also reported exposure and/or adverse effects data.

References cited	Countries covered	Street-based activities/industries	Exposures/agents of concern	Risks/adverse health effects or outcomes
Baron (2005) [60]	Mexico	Street children related to informal sector jobs (i.e. vending)	Physical exposure	Injuries (sprains, strains, fracture, deep lacerations, amputations, paralysis)

Bromley and Mackie (2009) [61]	Peru	Traders (selling and exchanging goods, foods, etc.)	Physical exposure (accident, abuse, theft)	Physical injuries

Furman and Laleli (2000) [62]	Turkey (Istanbul)	Vendors (selling goods, foods, etc.)	Lead	High hair lead concentration

Gharaibeh and Hoeman (2003) [63]	Jordan	Garage boys	Chemical fumes, paints; metal parts falling, cutting; cold hands, feet; abuses (physical, verbal and sexual)	Eye burning; hand injuries; injuries from heavy object(s) falling

Mondal et al. (2012) [64]	India	Shoe-polishers; vendors; performers; sweepers	N/A	RTI, eczema and pyoderma; diarrhea and abdomen pain

Pinzón-Rondóna et al. (2009, 2010) [53, 54]	Brazil, Colombia, Ecuador, Peru	Vendors; cleaners; car guarders; performers	Physical exposure	Injuries (scratches, cut, burn, car accident, sprains, amputations)

**Table 4:** Hazards/pollutants noted in papers reviewed by exposure agent categories of concern to public health.

Category	Hazards/pollutants
Biological	Infectious diseases transmitted by mosquitoes and parasites and via blood; HIV/AIDS

Chemical (organic)	Solvents used for cleaning and in tanning/leather industry and agricultural pesticides (i.e., mixtures of chemicals with active and inactive ingredients)

Chemical (inorganic)	Lead, mercury, arsenic, cadmium, chromium, traffic-related pollutants

Physical	Various ergonomic factors, for example, repetitive motions, postures; various sharp tools/objects of various sizes; hot and cold conditions both outdoors and indoors (including semienclosed microenvironments and underground mines)

Psychosocial (social and/or psychological)	Socioeconomic status indicators (maternal and/or paternal (parental) education; family income); physical abuse; verbal abuse; gender

Radiological	*None specifically reported*

## References

[1] United States Department of Labor (USDOL). International Labor Bureau (ILAB) http://www.dol.gov/ilab/projects/.

[2] USDOL-ILAB US Labor Department commemorates 20 years of work combating international child labor with release of new reporting and $26 million in grants. http://www.dol.gov/opa/media/press/ilab/ilab20132014.htm.

[3] Amon J. J., Buchanan J., Cohen J., Kippenberg J. (2012). Child labor and environmental health: government obligations and human rights. *International Journal of Pediatrics*.

[4] International Labour Organization (ILO) (2015) C138-Minimum Age Convention. http://www.ilo.org/dyn/normlex/en/f?p=NORMLEXPUB:12100:0::NO::P12100_ILO_CODE:C138.

[5] ILO http://www.ilo.org/dyn/normlex/en/f?p=NORMLEXPUB:12100:0::NO::P12100_ILO_CODE:C182.

[6] United Nations (1989). *Convention on the Rights of the Child*.

[7] Perera F. (2014). Science as an early driver of policy: child labor reform in the early progressive Era, 1870–1900. *American Journal of Public Health*.

[8] World Health Organization (WHO) About WHO: WHO regional offices. http://www.who.int/about/regions/en/.

[9] Mull L. D., Kirkhorn S. R. (2005). Child labor in Ghana cocoa production: focus upon agricultural tasks, ergonomic exposures, and associated injuries and illnesses. *Public Health Reports*.

[10] Otañez M. G., Muggli M. E., Hurt R. D., Glantz S. A. (2006). Eliminating child labour in Malawi: a British American Tobacco corporate responsibility project to sidestep tobacco labour exploitation. *Tobacco Control*.

[11] Kouvonen A., Lintonen T. (2002). Adolescent part-time work and heavy drinking in Finland. *Addiction*.

[12] Holgado D., Maya-Jariego I., Ramos I. (2014). Impact of child labor on academic performance: evidence from the program ‘Edúcame Primero Colombia’. *International Journal of Educational Development*.

[13] Castro C. L., Hunting K. (2013). Measuring hazardous work and identifying risk factors for non-fatal injuries among children working in Philippine agriculture. *American Journal of Industrial Medicine*.

[14] El-Gilany A.-H., Khalil A.-H., El-Wehady A. (2007). Epidemiology and hazards of student labour in Mansoura, Egypt. *Eastern Mediterranean Health Journal*.

[15] Castro C. L., Gormly S., Ritualo A. R. (2005). The SIMPOC Philippine survey of children 2001: a data source for analyzing occupational injuries to children. *Public Health Reports*.

[16] Anwer I., Awan J. A. (2003). Nutritional status comparison of rural with urban school children in Faisalabad District, Pakistan. *Rural and Remote Health Journal*.

[17] Tzannatos Z. (2003). Child labor and school enrollment in Thailand in the 1990s. *Economics of Education Review*.

[18] Waghamode R. H., Kalyan J. L. (2013). Child labour in India: an analytical approach. *Indian Streams Research Journal*.

[19] Srivastava K. (2011). Child labour issues and challenges. *Industrial Psychiatry Journal*.

[20] Fetuga B. M., Njokama F. O., Olowu A. O. (2005). Prevalence, types and demographic features of child labour among school children in Nigeria. *BMC International Health and Human Rights*.

[21] Admassie A. (2003). Child labour and schooling in the context of a subsistence rural economy: can they be compatible?. *International Journal of Educational Development*.

[22] de Vasconcelos R. B. A., Santos J. C. V., Araujo R. F., de Souza L., Dantas R. A. A., Gurgel R. Q. (2010). Occupational injuries in children and adolescents in emergency services of Aracaju, Brazil. *Child: Care, Health and Development*.

[23] Banerjee S. R. (1993). Agricultural child labor in West Bengal. *Indian Pediatrics*.

[24] Hendricks K. J. (2014). Youth on racial minority operated U.S. farms, 2008: demographics and injuries. *Journal of Safety Research*.

[25] Das B., Ghosh T., Gangopadhyay S. (2013). Child work in agriculture in West Bengal, India: assessment of musculoskeletal disorders and occupational health problems. *Journal of Occupational Health*.

[26] Corriols M., Aragón A. (2010). Child labor and acute pesticide poisoning in Nicaragua: failure to comply with children's rights. *International Journal of Occupational and Environmental Health*.

[27] Mitchell R. J., Franklin R. C., Driscoll T. R., Fragar L. J. (2001). Farm-related fatalities involving children in Australia, 1989–92. *Australian and New Zealand Journal of Public Health*.

[28] Chiavegatto C. V., Scalia Carneiro A. P., Costa Dias E., Nascimento M. S. (2010). Diagnosis of severe silicosis in young adults working in stone polishing and mining in Minas Gerais, Brazil. *International Journal of Occupational and Environmental Health*.

[29] Hilson G. (2008). ‘A load too heavy’: critical reflections on the child labor problem in Africa's small-scale mining sector. *Children and Youth Services Review*.

[30] Hilson G. (2012). Family hardship and cultural values: child labor in malian small-scale gold mining communities. *World Development*.

[31] Maconachie R., Hilson G. (2016). Re-thinking the child labor ‘problem’ in Rural sub-Saharan Africa: the case of sierra leone's half shovels. *World Development*.

[32] Saha A., Sadhu H. G. (2013). Occupational injury proneness in young workers: a survey in stone quarries. *Journal of Occupational Health*.

[33] Hosseinpour M., Mohammadzadeh M., Atoofi M. (2014). Work-related injuries with child labor in Iran. *European Journal of Pediatric Surgery*.

[34] Bessell S. (2011). Influencing international child labour policy: the potential and limits of children-centred research. *Children and Youth Services Review*.

[35] Joshi S. K., Dahal P. (2008). Occupational health in small scale and household industries in Nepal: a situation analysis. *Kathmandu University Medical Journal*.

[36] Saddik B., Nuwayhid I., Williamson A., Black D. (2003). Evidence of neurotoxicity in working children in Lebanon. *NeuroToxicology*.

[37] Saddik B., Williamson A., Nuwayhid I., Black D. (2005). The effects of solvent exposure on memory and motor dexterity in working children. *Public Health Reports*.

[38] Ide L. S. R., Parker D. L. (2005). Hazardous child labor: lead and neurocognitive development. *Public Health Reports*.

[39] Esin M. N., Bulduk S., Ince H. (2005). Work-related risks and health problems of working children in urban Istanbul, Turkey. *Journal of Occupational Health*.

[40] Nuwayhid I. A., Usta J., Makarem M., Khudr A., El-Zein A. (2005). Health of children working in small urban industrial shops. *Occupational and Environmental Medicine*.

[41] Fassa A. G., Facchini L. A., Mór Dall'Agnol M., Christiani D. C. (2005). Child labor and musculoskeletal disorders: the pelotas (Brazil) epidemiological survey. *Public Health Reports*.

[42] Dufort V. M., Kotch J. B., Marshall S. W., Waller A. E., Langley J. D. (1997). Occupational injuries among adolescents in Dunedin, New Zealand, 1990–1993. *Annals of Emergency Medicine*.

[43] Das P. K., Shukla K. P., Öry F. G. (1992). An occupational health programme for adults and children in the carpet weaving industry, Mirzapur, India: a case study in the informal sector. *Social Science and Medicine*.

[44] Joshi S. K., Sharma P., Sharma U., Sitaraman S., Pathak S. S. (1996). Peak expiratory flow rate of carpet weaving children. *Indian Pediatrics*.

[45] Choobineh A., Shahnavaz H., Lahmi M. (2004). Major health risk factors in Iranian hand-woven carpet industry. *International Journal of Occupational Safety and Ergonomics*.

[46] Awan S., Nasrullah M., Cummings K. J. (2010). Health hazards, injury problems, and workplace conditions of carpet-weaving children in three districts of Punjab, Pakistan. *International Journal of Occupational and Environmental Health*.

[47] Gürcanli G. E. (2009). Who is at fault? Third party and child injuries at construction sites in Turkey. *Safety Science*.

[48] Tiwari R. R. (2013). Eyestrain in working children of footwear making units of Agra, India. *Indian Pediatrics*.

[49] Mitra S. (1993). A study of the health conditions of child workers in a small scale leather industry in Calcutta. *British Journal of Industrial Medicine*.

[50] Harari R., Cullen M. R. (1995). Childhood lead intoxication associated with manufacture of roof tiles and ceramics in the Ecuadorian Andes. *Archives of Environmental Health*.

[51] Rohlman D. S., Nuwayhid I., Ismail A., Saddik B. (2012). Using epidemiology and neurotoxicology to reduce risks to young workers. *NeuroToxicology*.

[52] Ersado L. (2005). Child labor and schooling decisions in urban and rural areas: comparative evidence from Nepal, Peru, and Zimbabwe. *World Development*.

[53] Pinzón-Rondóna A. M., Koblinsky S. A., Hofferth S. L., Pinzón-Florez C. E., Briceno L. (2009). Work-related injuries among child street-laborers in Latin America: prevalence and predictors. *Revista Panamericana de Salud Publica*.

[54] Pinzón-Rondóna A. M., Botero J. C., Benson L., Briceno-Ayala L., Kanamori M. (2010). Workplace abuse and economic exploitation of children working in the streets of Latin American cities. *International Journal of Occupational and Environmental Health*.

[55] Whetten R., Messer L., Ostermann J. (2011). Child work and labour among orphaned and abandoned children in five low and middle income countries. *BMC International Health and Human Rights*.

[56] Levison D., Langer A. (2010). Counting child domestic servants in Latin America. *Population and Development Review*.

[57] Gamlin J., Camacho A. Z., Ong M., Hesketh T. (2015). Is domestic work a worst form of child labour? The findings of a six-country study of the psychosocial effects of child domestic work. *Children's Geographies*.

[58] Webbink E., Smits J., de Jong E. (2012). Hidden child labor: determinants of housework and family business work of children in 16 developing countries. *World Development*.

[59] Roggero P., Mangiaterra V., Bustreo F., Rosati F. (2007). The health impact of child labor in developing countries: evidence from cross-country data. *American Journal of Public Health*.

[60] Baron S. L. (2005). Injuries in child laborers in the informal sector in Mexico City, Mexico, 1997. *Public Health Reports*.

[61] Bromley R. D. F., Mackie P. K. (2009). Child experiences as street traders in Peru: contributing to a reappraisal for working children. *Children's Geographies*.

[62] Furman A., Laleli M. (2000). Semi-occupational exposure to lead: a case study of child and adolescent street vendors in Istanbul. *Environmental Research*.

[63] Gharaibeh M., Hoeman S. (2003). Health hazards and risks for abuse among child labor in Jordan. *Journal of Pediatric Nursing*.

[64] Mondal R., Sarkar S., Bhattacharya S. (2012). Profile of child labor in Indian railways. *Indian Journal of Pediatrics*.

[65] Kim C.-Y. (2009). Is combining child labour and school education the right approach? Investigating the Cambodian case. *International Journal of Educational Development*.

[66] Shafiq M. N. (2007). Household schooling and child labor decisions in rural Bangladesh. *Journal of Asian Economics*.

[67] Naeem Z., Shaukat F., Ahmed Z. (2011). Child labor in relation to poverty. *International Journal of Health Services*.

[68] Gjermeni E., Van Hook M. P., Gjipali S., Xhillari L., Lungu F., Hazizi A. (2008). Trafficking of children in Albania: patterns of recruitment and reintegration. *Child Abuse and Neglect*.

[69] Omokhodion F. O., Omokhodion S. I. (2004). Health status of working and non-working school children in Ibadan, Nigeria. *Annals of Tropical Paediatrics*.

[70] Schlick C., Joachin M., Briceño L., Moraga D., Radon K. (2014). Occupational injuries among children and adolescents in Cusco Province: a cross-sectional study. *BMC Public Health*.

[71] Invernizzi A. (2003). Street-working children and adolescents in Lima: work as an agent of socialization. *Childhood*.

[72] Estrada E., Hondagneu-Sotelo P. (2011). Intersectional dignities: latino immigrant street vendor youth in Los Angeles. *Journal of Contemporary Ethnography*.

[73] Rother H.-A. (2010). Falling through the regulatory cracks: street selling of pesticides and poisoning among Urban Youth in South Africa. *International Journal of Occupational and Environmental Health*.

[74] Hawamdeh H., Spencer N., Waterston T. (2001). Work, family socioeconomic status, and growth among working boys in Jordan. *Archives of Disease in Childhood*.

[75] Hawamdeh H., Spencer N. (2003). Effect of work related variables on growth among working boys in Jordan. *Journal of Epidemiology and Community Health*.

[76] Chaudhuri S. (2011). Labor market reform and incidence of child labor in a developing economy. *Economic Modelling*.

[77] Clark R. (2011). Child labor in the world polity: decline and persistence, 1980–2000. *Social Forces*.

[78] Parker D. L., Overby M. (2005). A discussion of hazardous child labor. *Public Health Reports*.

[79] Phillips N., Bhaskaran R., Nathan D., Upendranadh C. (2014). The social foundations of global production networks: towards a global political economy of child labour. *Third World Quarterly*.

[80] Doytch N., Thelen N., Mendoza R. U. (2014). The impact of FDI on child labor: insights from an empirical analysis of sectoral FDI data and case studies. *Children and Youth Services Review*.

[81] Estacio E. V., Marks D. F. (2005). Child labour and the international labour organization's Convention 182: a critical perspective. *Journal of Health Psychology*.

